# Corneal densitometry changes in a patient with interface fluid syndrome after small incision lenticule extraction

**DOI:** 10.1186/s12886-017-0428-0

**Published:** 2017-03-29

**Authors:** Ke Zheng, Tian Han, Meiyan Li, Yinan Han, Ye Xu, Rupal Shah, Xingtao Zhou

**Affiliations:** 1 Key Lab of Myopia, Ministry of Health, Department of Ophthalmology and Vision Science, Eye and ENT Hospital of Fudan University, No.19 Baoqing Road, Xuhui District, Shanghai, China; 2 New Vision Laser Centers, Vadodara, Gujarat India

**Keywords:** Small incision lenticule extraction (SMILE), Interface fluid syndrome (IFS), Corneal densitometry

## Abstract

**Background:**

To report a case of interface fluid syndrome (IFS) following small incision lenticule extraction (SMILE) evaluated with corneal densitometry and optical coherence tomography (OCT).

**Case presentation:**

An 18-year-old man reported sudden vision loss 24 days after SMILE procedure. Intraocular pressure (IOP) was 36.3 mmHg (OD) and 36.7 mmHg (OS) by noncontact tonometry. Moderate corneal edema, interface fluid pocket and haze were observed by OCT and confirmed by corneal densitometry values. Discontinuation of steroids and addition of hypotensive medication were offered immediately. The symptoms were cured after the medication. Changes of corneal densitometry were consistent with the clinical course of IFS.

**Conclusion:**

This case illustrates that it is crucial to be aware that a history of SMILE can also cause IFS. Both OCT and corneal densitometry can serve as auxiliary means to evaluate the clinical course of IFS, and appropriate IOP management is an effective approach.

## Background

Interface fluid syndrome (IFS) is characterized by accumulation of aqueous humor in intrastromal space. Most of reports on IFS have been described following laser in situ keratomileusis (LASIK) surgery [1, 2]. IFS mostly results from increased intraocular pressure (IOP) triggered by postoperative steroid administration [3, 4]. Rare causes of fluid accumulation include corneal endothelial cell decompensation [5–10]. IFS can be confused with diffuse lamellar keratitis (DLK), and misdiagnosis and inappropriate treatment can lead to irreversible visual field loss [4]. Moreover, IFS can occur a long time after LASIK, even 10 years afterwards [6, 7]. Thus, IFS should be kept in mind when prescribing steroids or performing other ocular surgery in patients with a history of LASIK.

Small incision lenticule extraction (SMILE) is a novel lamellar refractive surgery with a merely 2-mm incision. Flap-related complications following LASIK surgery are avoided in SMILE surgery, which helps to gain popularity of SMILE. However, the space between corneal cap and stromal bed still exists in SMILE, indicating the possibility of interface complications [11]. We recently observed a case of IFS following SMILE surgery.

IFS can decrease corneal transparency, which can be revealed by the increase of corneal densitometry [12, 13]. Thus, during the clinical course, anterior segment changes were observed using both optical coherence tomography (OCT) and an automated Scheimpflug densitometry program.

## Case presentation

An 18-year-old man underwent bilateral routine SMILE procedure with preoperative refraction of −6.00/–2.25x175 (OD) and −6.50/–1.75x180 (OS), and corrected distance visual acuity (CDVA) was 20/16 in both eyes. After the surgery, topical antibiotics (tobrex) and a non-preservative tear supplement were used. Topical steroids (fluorometholone 0.1%) were used initially eight times daily and tapered every three days. The patient ran out of fluorometholone 0.1% at 14 days postoperatively and local ophthalmologist prescribed him tobramycin-dexamethasone (tobradex) eyedrops three times daily for 4 days at 20 days postoperatively.

**Fig. 1:**
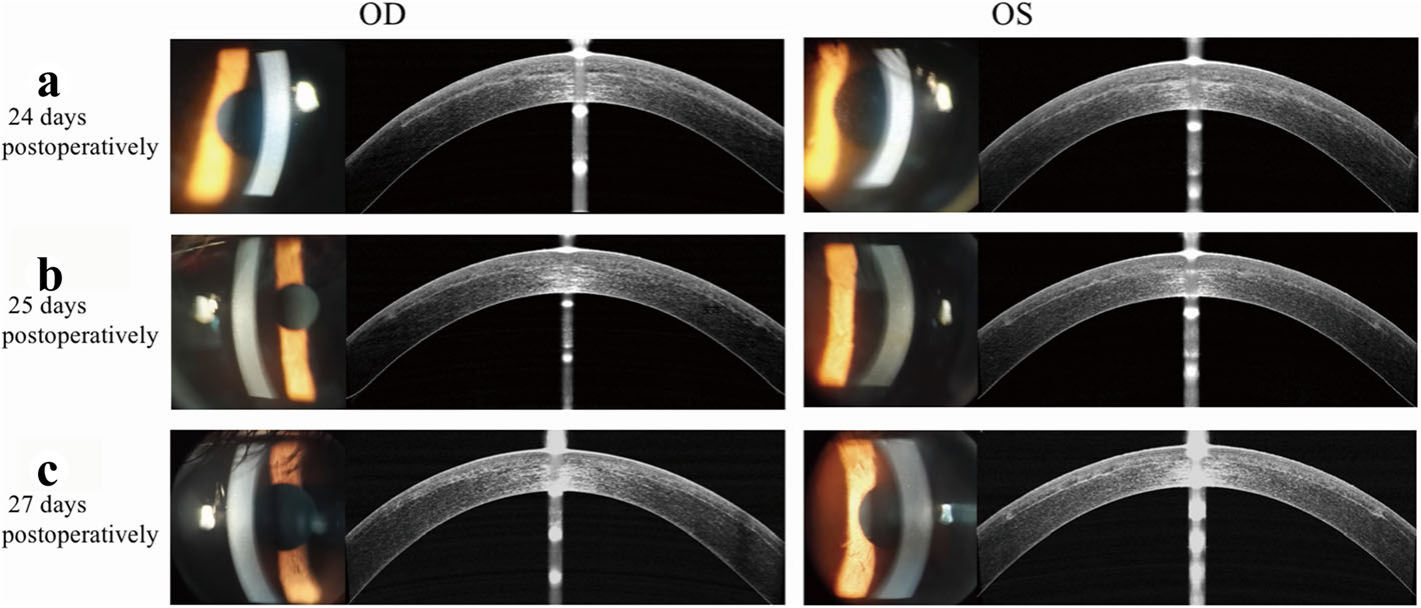
Representative slit-lamp and OCT photographs at (**a**) 24 days (**b**) 25 days (**c**)27 days postoperatively. **a** Slitlamp examination revealed interface haze and cornea edema. OCT showed interface fluid accumulation in the cap-bed interface of the both eyes. **b** Interface haze and cornea edema was relieved under slitlamp examination. OCT showed that interface fluid accumulation in the cap-bed interface of the both eye was also absorbed. **c** The cornea was clear under slitlamp examination. OCT showed merely mild microdistortions in Bowman’s layer

Then at 24 days postoperatively, the patient sought treatment for sudden vision loss. Uncorrected distance visual acuity (UDVA) was 20/80 in both eyes. Refraction was −3.75/–1.25x180 (OD) and −3.75/–0.50x180 (OS), and CDVA was 20/25 in both eyes. IOP was 36.3 mmHg (OD) and 36.7 mmHg (OS) by noncontact tonometry (NCT; Canon TX-20, Canon Corp., Japan). Slitlamp examination revealed interface haze and corneal edema, and fourier-domain optical coherence tomography (FD-OCT; RTVue, Optovue, Corp., USA) showed interface fluid accumulation in the cap-bed interface of both eyes (Fig. 1−a). Tobramycin-dexamethasone was stopped, an intravenous mannitol drip, carteolol hydrochloride eyedrops twice daily, pranoprofen eyedrops four times daily and 12.5 mg methazolamide twice daily were immediately administered. Eight hours after the treatment, IOP was 14.7 mmHg (OD) and 17.2 mmHg (OS). The next day, UDVA recovered to 20/25 in both eyes. Refraction was −2.00/–0.50x175 (OD) and −2.00/–0.50x10 (OS), and CDVA was 20/20 in both eyes. IOP was 16.1 mmHg (OD) and 16.7 mmHg (OS). Corneal endothelial densities measured by specular microscopy (Topcon SP-2000P, Topcon Corp., Japan) were 3800 cells/mm^2^ (OD) and 3232 cells/mm^2^ (OS). Interface haze and cornea edema were relieved under slitlamp examination. OCT showed that interface fluid accumulation in the cap-bed interface of both eyes was also absorbed (Fig. 1-b). The intravenous mannitol drip and methazolamide were discontinued, but pranoprofen and carteolol hydrochloride eyedrops were continued.

Two days later, UDVA was 20/20 in both eyes, with refraction of −0.50/–0.50x180 (OD) & -0.50/–0.25x15 (OS), and CDVA of 20/16 in both eyes. IOP was 11.2 mmHg (OD) and 11.7 mmHg (OS). The cornea was clear under slitlamp examination. OCT showed merely mild microdistortions in Bowman’s layer (Fig. 1−c). The carteolol hydrochloride eyedrops was discontinued, but pranoprofen eyedrops was continued. At 33 days postoperatively, his visual acuity and IOP remained stable in both eyes. Changes of corneal densitometry and central corneal thickness values using rotating Scheimpflug camera imaging (Pentacam HR 70900, OCULUS Corp., Germany) were shown in Table 1.

**Table 1:** Corneal densitometry and CCT Changes of a patient with IFS

Time	OD			OS		
	Anterior layer in total diameter	Central point (maximum)	CCT (μm)	Anterior layer in total diameter	Central point (maximum)	CCT (μm)
Pre-operation	19.2	28.2	523	20.3	25.9	520
24 days postoperatively (IFS was detected)	30.8	32.5	491	31.3	45.4	490
25 days postoperatively	23.9	30.6	440	23.0	31.8	434
26 days postoperatively	23.6	30.2	421	22.9	29.4	429
27 days postoperatively	20.7	26.7	410	21.9	27.8	409
33 days postoperatively	21.2	26.7	406	21.0	31.4	401

*IFS* Interface fluid syndrome

*CCT* Central corneal thickness

## Discussion and conclusions

Since Lyle and Jin [2] reported the first case of IFS after LASIK in 1999, the awareness of IFS has been promoted by increasing reports [4, 6, 7, 14]. As one of the lamellar refractive surgeries, SMILE generates space between corneal cap and stromal bed as LASIK, interface complications such as IFS can happen in SMILE [11]. This case is the first IFS case after SMILE procedure for the surgeon (XTZ) (incidence: nearly 1: 10000).

In this case, moderate corneal edema, interface fluid pocket and haze were observed, corresponding to stage 2 IFS according to Dawson’s grading system [1]. Dawson et al. [1] classified IFS into stages ranging from 1 to 3 based on the degree of fluid retention in the flap interface. IFS stage 2 sometimes can be confused with the slit-lamp appearance of DLK stage 1 or 2, but the treatments of the two diseases are totally distinct from each other. Misdiagnosis can cause optic nerve damage, irreversible visual field loss and central visual acuity decrease [4]. In addition, NCT has been shown to be dependent on central corneal thickness (CCT). In this case, NCT was underestimated due to decreased CCT and presence of interface fluid. It would be more accurate to measure IOP with other types of tonometry, especially dynamic contour tonometry, which may be less affected by CCT. Clinically, inaccurately low measurements of central IOP owing to cushioning of the fluid pocket can also confuse the diagnosis [15].

IFS was caused by elevated IOP in this case since the patient inappropriately applied tobramycin-dexamethasone three times for 4 days. Use of steroids is the most common reason of IFS [3, 4]. Other causes of IFS after LASIK are related to transient or permanent corneal endothelial cell decompensation in eyes with anterior uveitis [10], Fuchs endothelial dystrophy [8], traumatic hyphema [14] and eyes that have undergone certain procedures such as cataract surgery [7], vitreoretinal surgery [6], Descemet stripping automated endothelial keratoplasty (DSAEK) [5] or trabeculectomy [9]. These risk factors for interface fluid syndrome after LASIK should also be noted in post-SMILE eyes.

The refraction of the patient in this case changed from more than −3.00D to −0.75D during the clinical course, and the myopic shift influenced by IFS also indicated the condition of IFS.

Another interesting aspect of our case is the use of automated Scheimpflug densitometry program. Studies of corneal densitometry have attracted increasing interest over the past few years [12, 13]. Considering that IFS mainly affected the transparency of the anterior corneal layer, we show in Table 1 the densitometry of the total diameter of this layer and maximum central point. As shown in Table 1, the corneal densitometry increased at 24 days postoperatively. Corneal densitometry increases when edema, haze or inflammation occurs [12, 13]. According to our research, corneal densitometry declined to the baseline within a week after SMILE (J Refract Surg, 2017). Thus, in this case, the increase of corneal densitometry was mainly related to IFS. Moreover, the changes of corneal densitometry were consistent with the clinical course of IFS. Along with the recovery of IFS, corneal densitometry declined distinctly. Therefore, the corneal densitometry can be useful for evaluating and grading the condition of IFS quantitatively and objectively.

This case illustrates that it is crucial to be aware that a history of SMILE can also cause IFS. Both OCT and corneal densitometry can serve as auxiliary means to evaluate the clinical course of IFS, and appropriate IOP management is an effective approach.

## Abbreviations

ANOVA: Analysis of variance
CDVA: Corrected distance visual acuity
D: Diopters
HOAs: Higher-order aberrations
IQR: Interquartile range
LASIK: Laser in situ keratomileusis
PRK: Photorefractive keratectomy
QIRC: Quality of Life Impact of Refractive Correction
RMS: Root mean square
SE: Spherical equivalent
SMILE: Small incision lenticule extraction
SPSS: Statistical Package for Social Sciences
UDVA: Uncorrected distance visual acuity
